# Medicines use review service in community pharmacies in Spain: REVISA project

**DOI:** 10.1007/s11096-020-01158-2

**Published:** 2020-09-29

**Authors:** Nuria García-Agua Soler, Eugenia Gómez-Bermúdez, Vicente J. Baixauli-Fernández, Sara Bellver-Beltrán, Javier Velasco-Martínez, Antonio J. García Ruiz, Francisco Jódar-Sánchez

**Affiliations:** 1 Chair of Health Economics and Rational Use of Drugs, Department of Pharmacology. University of Málaga, Boulevard Louis Pasteur, 32, 29071 Málaga, Spain; 2 Pharmacoeconomics: Clinical and Economic Evaluation of Pharmaceutical Drugs and Palliative Care, Institute of Biomedical Research in Malaga (IBIMA), Málaga, Spain; 3 Illustrious in the Official College of Pharmacists of Málaga, Málaga, Spain; 4 Subcommittee Medicines Use Review, Spanish Society of Family and Community Pharmacy (SEFAC-Sociedad Española de Farmacia Familiar y Comunitaria), Madrid, Spain; 5 Group in Biomedical Informatics, Biomedical Engineering and Health Economy, Institute of Biomedicine of Seville, IBiS/Virgen del Rocío University Hospital/CSIC/University of Seville, Seville, Spain

**Keywords:** Adherence, Community pharmacies, Medicines use review, Polypharmacy, Willingness to pay

## Abstract

*Background* Community pharmacy services play an important role in controlling some factors related to medicine use and patients can benefit from these services to improve the adherence and knowledge of their medications, besides to reduce medicine-related problems. *Objective* The aim of the REVISA project is to carry out a study on preliminary implementation of the medicines use review service in Spanish community pharmacies. *Setting* Sixty-four community pharmacies from all regions of Spain. *Method* A preliminary implementation, cross-sectional multicentre study was conducted using a convenience sample of voluntary community pharmacies. A structured interview enabled to pharmacists to obtain a better understanding of patient’s medicines use. *Main outcome measure* Medicines use review-related time and cost, satisfaction and willingness to pay. *Results* A total of 495 patients were enrolled. The mean age of the patients was 66.1 years, with the majority females (56.4%) and a mean consumption of 5.7 medicines. A total of 2811 medicines were evaluated and 550 referral recommendations were made (29.8% to Primary Care). The mean time employed by the pharmacists in the medicines use review service was 52.8 min (medicines use review-related cost of €17.27). Most patients expressed a high level of satisfaction with this service (98.5%) and a willingness to pay for it (84%). *Conclusion* Medicines use review service in community pharmacies in Spain can be delivered, that it appears to be acceptable to patients and that most patients said they would be willing to pay for it. This service may offer an opportunity to promote inter-professional collaboration between pharmacists and general practitioners.

**Electronic supplementary material:**

The online version of this article (10.1007/s11096-020-01158-2) contains supplementary material, which is available to authorized users.

## Impacts on practice

- The medicines use review service appears to be acceptable to patients and the most of them would be willing to pay for this service.
- Patients can obtain extra help with their medications through the medicines use review service.
- The medicines use review service may offer an opportunity to promote inter-professional collaboration between pharmacists and general practitioners.

## Introduction

The ageing population, less healthy lifestyles and an increasing incidence of chronic conditions mean that multimorbidity is on the rise [1]. Major consequences of multimorbidity are functional impairment, poor quality of life, risk of mortality and high healthcare utilization and costs [2–4]. In addition, patients with multimorbidity are at higher risk of safety issues for many reasons, including polypharmacy, which may lead to poor medication adherence and adverse drug events [5–8].

Community pharmacy services play an important role in controlling some factors related to medicine use [9–12], and pharmaceutical services integrated with primary healthcare services are critical to achieve the desired outcomes and to significantly reduce harms that can otherwise arise from multiple medicine use, such as non-adherence or medicine-related problems [13].

Spain is among the European countries experiencing a major change toward an older population structure [14]. The intensity of the ageing process of the resident population in Spain is set to rise. In 2052, the group aged 64 years and over will account for 37% of the total population of Spain [15].

Primary health care is a whole-of-society approach to health and well-being centred on the needs and preferences of individuals, families and communities. Community pharmacists are the health professionals most accessible to the population and they maintain links with other health professionals in primary health care [16]. In Spain, A homogenous distribution of pharmacies has been achieved, with the average ratio of inhabitants to pharmacies among the lowest in Europe (2186 inhabitants/pharmacy) [17]. Hence, 87% of the Spanish population has a pharmacy less than 250 m from their homes, which implies that patients are regular customers and can benefit from services to improve the adherence and knowledge of their medications, besides to reduce medicine-related problems.

During the last years, the Spanish community pharmacies have evolved in their approach to the patient, actively engaging in the services they provide. The Spanish Society of Family and Community Pharmacy (*SEFAC, Sociedad Española de Farmacia Familiar y Comunitaria*) and the General Pharmaceutical Council of Spain (*Consejo General de Colegios Oficiales de Farmacéuticos*) have been developing strategies that allow the introduction of a new model of professional practice, based on a greater commitment and multidisciplinary cooperation, for the benefit of the patient [17, 18].

The REVISA project managed by Subcommittee Medicines Use Review (MUR) of SEFAC consisted in the development of an intervention protocol based on the official guidance of MUR service by the National Health Service. The MUR in England involves the pharmacist reviewing the patient’s use of their medication, ensuring they understand how their medicines should be used and why they have been prescribed, identifying any problems and then, where necessary, providing feedback to the prescriber [19].

## Aim of the study

The aim of the REVISA project is to carry out a study on preliminary implementation of the MUR service in Spanish community pharmacies. This aim is divided into two objectives: (1) to evaluate the implementation of the MUR service; (2) to evaluate the outcomes of the MUR service.

## Ethics approval

The protocol, and participant information and consent document were submitted to the Málaga Regional Research Ethics Committee, who approved the study (reference number 02/2016-PIR12). All patients signed an informed consent before their inclusion in the study.

## Method

### Study design

A preliminary implementation, cross-sectional multicentre study was conducted using a convenience sample of voluntary community pharmacies from all regions of Spain. Volunteers’ patients were invited by the pharmacists.

The study subjects were patients older than 18 years who signed informed consent and belonged to one of the following groups: (1) they were users of complex medicines (drugs with a device with difficulty manipulating, such as inhalers or self-injections; or even complex administration: extemporaneous preparations, ophthalmic or otic drug administration, rectal or vaginal administration or transdermal drug) [20]; (2) they were users of high-risk medicines (drugs that, when used incorrectly, can cause serious damage or even death to patients, such as amiodarone, dronedarone, digoxin, spironolactone, eplerenone or methotrexate) [21]; (3) and they were polypharmacy patients (patients with five or more prescribed medicines). The following groups were excluded from the study: (1) pregnant or lactating women; (2) individuals undergoing treatment with chemotherapy or radiotherapy; (3) individuals with physical or mental disabilities; (4) individuals who had undergone a medication review in the past year; and (5) individuals whose medication was subject to a delay in pick-up from the community pharmacy by their caregivers or family members.

### REVISA project

Our preliminary implementation study was developed in two phases: (1) the enrolment of community pharmacists who voluntarily and selflessly (there was no payment or other incentive) participated and trained in the MUR service. The training consisted of theoretical and practical online sessions and in-person, hands-on sessions. The theoretical phase included the visualization of videos and multiple-choice test about standard working procedure and on the data recording system for the project, and on a review of the most prevalent diseases (diabetes, COPD, pain, etc.). The practical phase developed with role-play of clinical cases and skills evaluation of the MUR service.

Community pharmacists who successfully passed (test and clinical cases approved) this phase could start phase two; (2) the enrolment of a minimum of 8 patients in community pharmacies and provision of the MUR service. The MUR service was provided at a structured interview with the patient to know and to revise their medicines use according on protocol based on the official guidance of MUR service by the National Health Service (page 1 of Appendix A).

### Variables

Three types of data were collected:

#### Data about patients at baseline to describe their sociodemography, medicines-use problems and health status

- Sociodemography characteristics of the patients.
- Medicines-use problems were measured using a non-validated questionnaire based on the official guidance of MUR service (page 1 of Appendix A).
- Health status: medicine use, adherence and health-related quality of life were analysed.
  - Medicine use was measured using the Anatomical, Therapeutic, Chemical classification system.
  - Adherence to the medicines was measured using the Morisky-Green test [22]. This test was administered to each patient for each of the prescribed medications. The variable was categorized as adherent or non-adherent; patients were considered non-adherent when they were non-adherent to any one of their medications.
  - Health-related quality of life was measured using the Spanish version of the EuroQol-5D-5L [23]. This generic questionnaire describes the health status along five dimensions (mobility, personal care, usual activities, pain/discomfort and anxiety/depression) and contains a visual analogue scale.

#### Data to describe the implementation of the MUR service documenting pharmacist’s processes and interventions

- Interventions to provide information/recommendations to the patients regarding the use of medicines.
- Inter-professional collaboration based on referrals recommendations to other health professionals or pharmaceutical services.

#### Data to evaluate the outcomes of the MUR service

- MUR-related time and cost: The time devoted to interviews with patients and registering the MUR form and reports were considered as the time associated with the MUR service. The unit cost of the community pharmacist was calculated taking into account the pharmacist’ salary in the Spanish community pharmacy agreement [24], and on the time the pharmacist devoted to the MUR service.
- Satisfaction and willingness to pay: To evaluate satisfaction with the MUR service, a validated satisfaction questionnaire was used to evaluate the pharmaceutical care service provided in community pharmacies [25]. To determine the patient’s willingness to pay, they were asked whether they would use the MUR service again and, if so, the amount they would be willing to pay for it. The response options were structured in closed ranges from 0 euros to more than 30 euros. Both questionnaires were anonymous.

### Statistical analysis

A descriptive analysis was performed using absolute and relative frequencies for qualitative variables, and mean ± standard deviation for quantitative variables. Comparisons between variables were performed using bivariate analysis. The relationship between quantitative variables was analysed through Pearson´s correlation coefficient. The estimate of the magnitude of the relationship between variables was analysed using the odds ratio (OR) and 95% confidence interval (95% CI) by a logistic regression analysis. The data were analysed using the SPSS 20.0 statistical program for Windows.

## Results

### Sociodemography characteristics, medicines-use problems and health status

#### Patients characteristics

Sixty-four community pharmacies participated, most of them were suburban pharmacies (59.4%), followed by rural (20.3%), transit (17.2%) and coastal (3.1%) pharmacies. A total of 495 patients were enrolled. Table 1 shows the sociodemographic characteristics of the participating patients. A slight predominance of women (56.4%) was noted, with a mean age of 66.09 ± 14.71 years and a mean consumption of 5.68 ± 2.97 medicines. Additionally, 62.2% of the patients were aged 65 years or older, 61.2% were polypharmacy patients (although only 45.3% of patients met both conditions), 10.1% were patients with complex medicines and 33.9% were patients with high-risk medicines.

**Table 1:** Patient sociodemographic characteristics

Variable	Categories	Value
Gender	Female	279 (56.4)
	Male	216 (43.6)
Age in years	Mean + SD	66.1 ± 14.7
	< 65	187 (37.8)
	≥ 65	308 (62.2)
Marital status^a^	Single	50 (10.1)
	Married	310 (62.6)
	Separated/divorced	37 (7.5)
	Widow	94 (19)
Education level^a^	None	92 (18.6)
	Elementary	183 (37)
	Secondary	104 (21)
	University	102 (20.6)
	Other	3 (0.6)
Occupation^a^	Employed	91 (18.4)
	Unemployed	31 (6.3)
	Retired	267 (53.9)
	Homemaker	67 (13.5)
	Other	14 (2.8)
Cohabitation^b^	Alone	81 (16.4)
	Partnered	273 (55.2)
	Children	99 (20)
	Caregiver—part-time	13 (2.6)
	Caregiver—full-time	7 (1.4)
	Other	30 (6.1)
Number of medicines prescribed	Mean + SD	5.68 ± 2.97
	≤ 4	192 (38.8)
	5–9	250 (50.5)
	10–14	46 (9.3)
	≥ 15	7 (1.4)
Help with medication^a^	None	397 (80.2)
	Children	25 (5.1)
	Caregiver	13 (2.6)
	Others	20 (4.0)
Country of origin^a^	Spain	481 (97.2)
	Other	12 (2.4)

Data are expressed as n (%) or mean ± standard deviation

^a^There are missing values for these variables

^b^Answers were not mutually exclusive

#### Medicines-use problems

Table 2 shows the medicines-use problems.

**Table 2:** Medicines-use problems

	Yes	No	DK
Inappropriate dose	146 (5.2)	2617 (93.1)	48 (1.7)
Inappropriate pattern	325 (11.6)	2445 (87.0)	41 (1.4)
Inappropriate duration	125 (4.5)	2643 (94.0)	43 (1.5)
Suboptimal administration	203 (7.2)	2563 (91.2)	45 (1.6)
Inappropriate conservation	115 (4.1)	2640 (93.9)	56 (2.0)
Inappropriate disposal	273 (9.7)	2415 (85.9)	123 (4.4)
Difficulty with use	110 (3.9)	2651 (94.3)	50 (1.8)
Concerns about use	193 (6.9)	2548 (90.6)	70 (2.5)
Patient does not know the drug indication	289 (10.3)	2446 (87.0)	76 (2.7)
Suboptimal treatment of illness	170 (6.1)	2550 (90.7)	91 (3.2)
Inappropriate dose or duration (deliberate)	179 (6.4)	2447 (87.0)	185 (6.6)
Suspected adverse drug reactions	246 (8.8)	2516 (89.5)	49 (1.7)
Patient requests further information	176 (6.3)	2584 (91.9)	51 (1.8)
Duplicity	44 (1.6)	2688 (95.6)	79 (2.8)
Contraindication	20 (0.7)	2714 (96.6)	77 (2.7)
Interactions	124 (4.4)	2601 (92.5)	86 (3.1)
Other	116 (4.1)	2492 (88.7)	203 (7.2)

Data are expressed as n (%). DK: don’t know

#### Health status: medicines use, adherence to the medicines and health-related quality of life

In total, 2811 medicines were evaluated, over 90% were from the following six groups of the first Anatomical, Therapeutic, Chemical classification system (anatomical level): A)Alimentary tract and metabolism: 495 medicines (17.6%); B)Blood and blood-forming organs: 230 medicines (8.2%); C)Cardiovascular system: 900 medicines (32%); M)Musculo-skeletal system: 157 medicines (5.6%); N)Nervous system: 566 medicines (20.1%); R)Respiratory system: 210 medicines (7.5%).

Adherence for the 2811 medicines was analysed. Some patients claimed they had never forgotten to take their medication (80.6%), took it at the right times (88.4%) and did not stop taking it even if they felt well (87.2%) or felt ill (93.4%). Therefore, the patients were adherent for 68.3% of their medicines. Differences were found according to the Anatomical, Therapeutic, Chemical classification system: A (72.4%); B (80.3%); C (74.3%); M (60%); N (65%); R (64.3%); for the drugs in other groups: 73.8% (*p* < 0.001).

At the patient level, only 156 patients (31.5%) were adherent. Polypharmacy was associated with non-adherence (OR = 1.34; 95% CI: 0.91–1.97). Likewise, among polypharmacy patients older than 65 years, the degree of non-adherence was higher (73.7%) than that of the remaining patients (64.2%); (OR = 1.56; 95% CI: 1.06–2.30).

At least half of the patients reported having no health problems on most of the dimensions of the EuroQol-5D-5L: 58.4% for mobility, 77.0% for personal care, 68.7% for usual activities, 38.6% for pain/discomfort and 56.2% for anxiety/depression. The mean visual analogue scale score was 66.06 ± 17.81. Health-related quality of life reduced significantly for polypharmacy (63.39 ± 17.72 vs 70.25 ± 17.17; *p* < 0.001) and age older than 65 years (64.59 ± 17.53 vs 68.57 ± 18.06; *p* = 0.02). No differences were detected according non-adherence with the medication (65.62 ± 17.48 vs 67.01 ± 18.53; *p* = 0.45).

### Implementation of the MUR service documenting pharmacist’s processes and interventions

The pharmacists provided tailored information for 2073 medicines (73.8%) and 1316 suggestions for improving use (46.8%). At the patient level, the pharmacists provided personalized information to 473 patients (95.6%), suggestions for improvement to 423 patients (85.5%) and basic health education information to 417 patients (84.2%).

A total 550 referral recommendations were made in 334 patients: 164 (29.8%) to Primary Care, 19 (3.5%) to Specialized Care and the remaining referrals were to professional pharmaceutical services: 154 (28%) to the blood pressure monitoring service; 56 (10.2%) to the nutritional status assessment service; 55 (10%) to the personalized medication dosage systems service; 42 (7.6%) to the pharmaceutical care service with follow-up; 31 (5.6%) to the smoking cessation service; and 29 (5.3%) to other pharmaceutical services. Fourteen notifications were also made using the “yellow card” system [26].

Non-adherence to the medication and polypharmacy were the main factors associated with referral recommendations to healthcare and pharmaceutical services (Table 3). In particular, non-adherence to the medication (OR = 1.84; 95% IC: 1.20–2.82) and polypharmacy (OR = 1.64; 95% IC: 1.11–2.44) were associated with recommendations for referrals to Primary Care.

**Table 3:** Predictors of referral recommendations

Factor	OR (95% CI)	*P* value
*Healthcare and pharmaceutical services*		
Non-adherence	1.74 (1.17–2.58)	0.006
Polypharmacy	1.50 (1.02–2.20)	0.038
*Primary care*		
Non-adherence	1.84 (1.20–2.82)	0.005
Polypharmacy	1.64 (1.11–2.44)	0.013
*Specialized care*		
Non-adherence	2.53 (0.73–8.80)	0.132
Polypharmacy	0.87 (0.34–2.19)	0.76
*Pharmaceutical services*		
Non-adherence	1.38 (0.94–2.01)	0.10
Polypharmacy	1.41 (0.98–2.02)	0.065

OR: odds ratio; 95% CI: 95% confidence interval

### Outcomes of the MUR service

#### MUR-related time and costs

The mean time employed by the pharmacists in the MUR was 52.80 ± 31.52 min: 27.34 ± 15.15 in the interview and 25.39 ± 21.32 for registering the MUR forms and reports.

There was a significant correlation between the MUR time and number of medicines (r = 0.54; *p* < 0.001). MUR-related time increased significantly for polypharmacy patients (62.27 ± 33.92 vs 37.98 ± 19.77; *p* < 0.001), patients aged 65 years or older (55.57 ± 32.95 vs 48.17 ± 28.48; *p* = 0.013) and non-adherent patients (54.94 ± 33.29 vs 48.17 ± 26.84; *p* = 0.029).

The mean costs were €8.98 ± 4.99 (associated with the interview) and €8.29 ± 7.01 (associated with MUR form registration), resulting in a mean MUR-related cost of €17.27 ± 10.31.

#### Satisfaction and willingness to pay

98.5% of patients expressed a high level of satisfaction with the MUR service (68.9% and 29.5% of patients were very satisfied and satisfied respectively). Among the main benefits of the MUR service that patients highlighted were better understanding of the medicines used (97.2%), effective resolution of health problem(s) (93.6%), learning the need to comply with the prescribed treatment (91.0%), learning the undesirable effects of the drugs used (87.7%), and reducing the undesirable effects of the drugs used (83.6%).

90.7% of patients indicated that they would likely use the service again, 8.4% of patients were undecided, and only 0.9% stated that they were unlikely to use the service again. Moreover, 91.1% of patients would recommend the service without hesitation, 7.8% would recommend it with reservations, and only 1.1% would not recommend it.

Four hundred nineteen patients expressed interest in receiving the MUR service. The willingness to pay was: 17 patients (4.1%) more than €30; 35 patients (8.4%) between €21 and €30; 91 patients (21.7%) between €11 and €20; 113 patients (27.0%) between €6 and €10, 96 patients (22.9%) until €5; 61 patients (14.6%) were not willing to pay anything for it; and 6 patients (1.4%) don´t know.

## Discussion

Our study presents initial findings on a preliminary implementation of the MUR service through pharmacist-led initiatives to obtain a better understanding of patients’ medicines use and their adherence to the medicines.

The results of this study suggest that patients can obtain extra help with their medications through the MUR service. Non-adherence and poor knowledge about medicines are behaviours that intensify with age and polypharmacy, indicating the target population for which MUR can be most effective [27–32].

The REVISA project has proven useful for patients given the high number of interventions that have occurred, which provided personalized information about medicines, suggestions regarding their use, health education and referrals to various health professionals, in particular general practitioners. MUR service may offer an opportunity to promote inter-professional collaboration between pharmacists and general practitioners. However, it has been reported that some of the problems with MUR have been that they do not integrate well with the patients’ Primary Care pathway, and that physicians s are commonly not particularly positive about this service [33–36]. In addition, a lack of communication and collaboration between general practitioners and community pharmacists can have an impact on the relationship between the patients and their general practitioners [37, 38].

Our results show that the MUR service appears well received by patients, even if an additional cost would be involved for the individual patient. Patients reported a high degree of satisfaction with the MUR service, in accordance with previous studies [37, 39, 40]. However, evidence that patients do not necessarily see a role of community pharmacy in delivering services that go much beyond their traditional supply and related advice function is also available [41].

The feasibility of incorporating this service into everyday practice would need to be assessed. Few studies have assessed the time invested in this service, which is an important factor considering the cost involved. Lee et al. [42] obtained a median time required for pharmacists to perform the initial MUR visit very similar to our results, although a scoping review of the medicines use review reported that the MUR consultations were short, typically 10–12 min [43]. Our results show that there was a significant correlation between the MUR time and number of medicines. Polypharmacy, age and non-adherence were other factors that significantly increased the MUR time.

Reforms to the service suggest that the MURs are becoming more responsive to patients’ need and preferences [44]. Our findings suggest that MUR service appears to be acceptable to patients and that most patients said they would be willing to pay for it.

There were some limitations to the present study. Non-random selection of study community pharmacies and patients limits generalizability of the results, although the participating community pharmacies represented all regions of Spain. A high degree of satisfaction with the services was obtained. However, it should be kept in mind that patient satisfaction with community pharmacy services, which they have agreed to participate in, is usually high. The time employed by the pharmacists in the MUR was high and it can be explain by the high percentages of polypharmacy patients and patients with complex medicines. In addition, the effectiveness of the referrals to health professionals is unclear, since there was no follow-up. Finally, this is a preliminary implementation study and its design is weak for an intervention evaluation. Therefore, it would be necessary to be able to continue with the REVISA project, and to carry out an intervention evaluation in order to provide the benefits of the MUR service in patients.

## Conclusion

The MUR service in community pharmacies in Spain can be delivered, that it appears to be acceptable to patients and that most patients said they would be willing to pay for it. The MUR service may offer an opportunity to promote inter-professional collaboration between pharmacists and general practitioners. Pharmacists self-reported the length of time taken to deliver a MUR although the feasibility of incorporating this service into everyday practice would need to be assessed.

## Electronic supplementary material

Below is the link to the electronic supplementary material.

Supplementary material 1 (PDF 348 kb)
