# Supplementary material for: Medicines use review service in community pharmacies in Spain: REVISA project
Source: Int J Clin Pharm. 2020 Sep 29;43(3):524–31. doi: 10.1007/s11096-020-01158-2 (PMC8214585; doi:10.1007/s11096-020-01158-2)
Supplement: Supplementary file 1 — Supplementary material 1 (PDF 348 kb) [file 11096_2020_1158_MOESM1_ESM.pdf]

MUR SERVICE

DATE OF REVIEW: .... **MUR Number:** ....

PHARMACIST INFORMATION

Pharmacy: ....
Address: ....
Telephone: ....
e-mail: ....
Pharmacist name and surname: ....
Registration number: ....

PATIENT INFORMATION

Name and surname: ....
Identification code: .... Identification card: .... Date of birth: ....
Telephone: .... e-mail: ....
Allergies: ....
☐ Consent form signed ☐ MUR with caregiver

Greatest health concern: ....

revisa

Sociedad Española de Farmacia Familiar y Comunitaria
SEFAC

How are you getting on with your medication? Is there anything you are worried about?	How do you take/use your medication?	Are you having any problems with your medicines or concerns about taking/using them?	Have you ever missed any dose of your medicines? (1)	Do you know why or what you are taking this medicine for?	Do you think it's causing any adverse or unexpected effects?	Is there anything else you'd like to know about your medication?	Incidents identified
	The pharmacist will check if the prescribed dosage is what the patient is taking: dose, pattern, duration; before or after food?splits tablet?; prepare/administer right?<br>The pharmacist will check if: conservation/disposal		Do you take the medication at the prescribed times? (2)<br>If you forget to take them, what do you do?	Do you think it's working? When you feel well, do you stop taking them? (3)	When you feel unwell, do you stop taking them? (4)	Patient requests further information	
**Name/Code medicine:**		**Dosage:**	**For(illness):**		**From:**	**Till:**	**Indicated by:*** ....
☐ He's not doing Well<br>*Notes***:	☐ Inappropriate dose ....<br>☐ Inappropriate pattern ....<br>☐ Inappropriate duration ....<br>☐ Suboptimal administration ....<br>☐ Inappropriate conservation..........<br>☐ Inappropriate disposal	☐ Difficulty in use**<br>☐ Use concern	☐ Non-compliance according to Morisky-Green test (answer "yes" on questions 1,3,4).<br>*Notes***:	☐ Does not know what he/she is using the medicine for<br>☐ Suboptimally treated illness	☐ Side effect**	☐ Doubts: Ask for information**	☐ Duplicity<br>☐ Contraindication<br>☐ Interactions<br>☐ Others (indicate):
**Name/Code medicine:**		**Dosage:**	**For(illness):**		**From:**	**Till:**	**Indicated by:*** ....
☐ He's not doing Well<br>*Notes***:	☐ Inappropriate dose ....<br>☐ Inappropriate pattern ....<br>☐ Inappropriate duration ....<br>☐ Suboptimal administration ....<br>☐ Inappropriate conservation..........<br>☐ Inappropriate disposal	☐ Difficulty in use**<br>☐ Use concern	☐ Non-compliance according to Morisky-Green test (answer "yes" on questions 1,3,4).<br>*Notes***:	☐ Does not know what he/she is using the medicine for<br>☐ Suboptimally treated illness	☐ Side effect**	☐ Doubts: Ask for information**	☐ Duplicity<br>☐ Contraindication<br>☐ Interactions<br>☐ Others (indicate):
**Name/Code medicine:**		**Dosage:**	**For(illness):**		**From:**	**Till:**	**Indicated by:*** ....
☐ He's not doing Well<br>*Notes***:	☐ Inappropriate dose ....<br>☐ Inappropriate pattern ....<br>☐ Inappropriate duration ....<br>☐ Suboptimal administration ....<br>☐ Inappropriate conservation..........<br>☐ Inappropriate disposal	☐ Difficulty in use**<br>☐ Use concern	☐ Non-compliance according to Morisky-Green test (answer "yes" on questions 1,3,4).<br>*Notes***:	☐ Does not know what he/she is using the medicine for<br>☐ Suboptimally treated illness	☐ Side effect**	☐ Doubts: Ask for information**	☐ Duplicity<br>☐ Contraindication<br>☐ Interactions<br>☐ Others (indicate):
**Name/Code medicine:**		**Dosage:**	**For(illness):**		**From:**	**Till:**	**Indicated by:*** ....
☐ He's not doing Well<br>*Notes***:	☐ Inappropriate dose ....<br>☐ Inappropriate pattern ....<br>☐ Inappropriate duration ....<br>☐ Suboptimal administration ....<br>☐ Inappropriate conservation..........<br>☐ Inappropriate disposal	☐ Difficulty in use**<br>☐ Use concern	☐ Non-compliance according to Morisky-Green test (answer "yes" on questions 1,3,4).<br>*Notes***:	☐ Does not know what he/she is using the medicine for<br>☐ Suboptimally treated illness	☐ Side effect**	☐ Doubts: Ask for information**	☐ Duplicity<br>☐ Contraindication<br>☐ Interactions<br>☐ Others (indicate):

(1)(2)(3)(4): Morisky-Green test questions. * Doctor (Dr), Nurse (N), Pharmacist (P), self-medication (SM). ** Give details with more information.

© Spanish Society of Family and Community Pharmacy (SEFAC). All rights saved. The content of this work is protected by Law, which establishes prison sentences and / or fines, in addition to the corresponding compensation for damages and prejudices, for those who reproduce, plagiarize, distribute or communicate publicly, the whole or one part, a literary, artistic or scientific work, or its transformation, interpretation or artistic execution fixed on any type of support or communicated through any means, without the required written authorization of the Copyright holders.

MUR SERVICE

DATE OF REVIEW: .... **MUR Number:** ....

PHARMACIST INFORMATION

Pharmacy: ....
Address: ....
Telephone: ....
e-mail: ....
Pharmacist name and surname: ....
Registration number: ....

PATIENT INFORMATION

Name and surname: ....
Identification code: .... Identification card: .... Date of birth: ....
Telephone: .... e-mail: ....
Allergies: ....
☐ Consent form signed ☐ MUR with caregiver
Greatest health concern: ....

revisa

Sociedad Española de Farmacia Familiar y Comunitaria

SEFAC

INTERVENTIONS

☐ Provide tailored information about the medication:

....

☐ Provide basic information about healthy routines: vaccination, healthy lifestyle (food and sport), illness.
Other:

....

☐ Provide support and advice to improve the use of the medication

....

☐ Doctor referral
Indicate the medicine referred for. Add all the information needed:

....

☐ Other healthcare professional referral.
Indicate the number of medicine referred and the professional. Add all the information needed.

....

☐ Refer to other service in the community pharmacy:
- ☐ Pharmacological treatment follow up
- ☐ Smoking cessation
- ☐ MDS
- ☐ Blood pressure monitoring
- ☐ Nutritional status assessment
- ☐ Health education
- ☐ Others: ....

☐ Notification via “yellow card”

☐ Others:

....

☐ Time spent during MUR: ....

☐ Time spent during registration: ....

Notes

....

....

....

....

....

....

....

....

....

....

....

© Spanish Society of Family and Community Pharmacy (SEFAC). All rights saved. The content of this work is protected by Law, which establishes prison sentences and / or fines, in addition to the corresponding compensation for damages and prejudices, for those who reproduce, plagiarize, distribute or communicate publicly, the whole or one part, a literary, artistic or scientific work, or its transformation, interpretation or artistic execution fixed on any type of support or communicated through any means, without the required written authorization of the Copyright holders.

MUR SERVICE

DATE OF REVIEW: .... **MUR Number:** ....

PHARMACIST INFORMATION

Pharmacy: ....
Address: ....
Telephone: ....
e-mail: ....
Pharmacist name and surname: ....
Registration number: ....

PATIENT INFORMATION

Name and surname: ....
Identification code: .... Identification card: .... Date of birth: ....
Telephone: .... e-mail: ....
Allergies: ....
☐ Consent form signed ☐ MUR with caregiver
Greatest health concern: ....

revisa

Sociedad Española de Farmacia Familiar y Comunitaria

SEFAC

PATIENT INFORMATION SHEET

....
....
....
....
....
....
....
....
....
....
....
....
....
....
....
....

© Spanish Society of Family and Community Pharmacy (SEFAC). All rights saved. The content of this work is protected by Law, which establishes prison sentences and / or fines, in addition to the corresponding compensation for damages and prejudices, for those who reproduce, plagiarize, distribute or communicate publicly, the whole or one part, a literary, artistic or scientific work, or its transformation, interpretation or artistic execution fixed on any type of support or communicated through any means, without the required written authorization of the Copyright holders.

MUR SERVICE

DATE OF REVIEW: .... **MUR Number:** ....

PHARMACIST INFORMATION

Pharmacy: ....
Address: ....
Telephone: ....
e-mail: ....
Pharmacist name and surname: ....
Registration number: ....

PATIENT INFORMATION

Name and surname: ....
Identification code: .... Identification card: .... Date of birth: ....
Telephone: .... e-mail: ....
Allergies: ....
☐ Consent form signed ☐ MUR with caregiver
Greatest health concern: ....

revisa

Sociedad Española de Farmacia Familiar y Comunitaria
SEFAC

REPORT Nº ....

These medicines have been checked:

☐ Review of medicines prescribed by the NHS and private providers
☐ Review of medicines and health products purchased OTC or self-prescribed
☐ Review of the degree of knowledge of the patient's medication and its indication
☐ Review of the posology (dosage, pattern and treatment duration)
☐ Review of the administration, conservation and disposal of the medication
☐ Provision of tailored information regarding the use of each of these medicines
☐ Detection of Medicine-Related Problems and/or Negative Outcomes of the medication
☐ Correction of errors, doubts and beliefs about the medication
☐ Provision of materials that support the patient in understanding and managing their medication and condition
☐ Provision of healthy lifestyle advice
☐ Referral to GP and/or other HCP

....

☐ Referral to other pharmaceutical services

....

☐ Actions agreed with patient, improvement proposals and recommendations (in more detail on the Patient Information Sheet of the MUR form)

Pharmacist signature

Signed: ....

© Spanish Society of Family and Community Pharmacy (SEFAC). All rights saved. The content of this work is protected by Law, which establishes prison sentences and / or fines, in addition to the corresponding compensation for damages and prejudices, for those who reproduce, plagiarize, distribute or communicate publicly, the whole or one part, a literary, artistic or scientific work, or its transformation, interpretation or artistic execution fixed on any type of support or communicated through any means, without the required written authorization of the Copyright holders.
